# ﻿Genome-wide survey reveals the phylogenomic relationships of *Chirolophisjaponicus* Herzenstein, 1890 (Stichaeidae, Perciformes)

**DOI:** 10.3897/zookeys.1129.91543

**Published:** 2022-11-11

**Authors:** Lu Liu, Qi Liu, Tianxiang Gao

**Affiliations:** 1 Naval Architecture and Port Engineering College, Shandong Jiaotong University, Weihai, China Shandong Jiaotong University Weihai China; 2 Wuhan Onemore-tech Co., Ltd. Wuhan, Hubei, China Wuhan Onemore-tech Co., Ltd Wuhan China; 3 Fishery College, Zhejiang Ocean University, Zhoushan, Zhejiang, China Zhejiang Ocean University Zhoushan China; 4 Zhejiang Provincial Key Laboratory of Mariculture and Enhancement, Zhejiang Marine Fisheries Research Institute, Zhoushan, China Zhejiang Marine Fisheries Research Institute Zhoushan China

**Keywords:** Chirolophinae, draft genome, genome assembly, genome evolution, next-generation sequencing, Stichaeidae, Zoarcales

## Abstract

Fish are the largest vertebrate group, consisting of more than 30 000 species with important ecological and economical value, while less than 3% of fish genomes have been published. Herein, a fish, *Chirolophisjaponicus*, was sequenced using the next-generation sequencing. Approximately 595.7 megabase pair of the *C.japonicus* genome was assembled (49 901 contigs with 42.61% GC contents), leading to a prediction of 46 729 protein-coding gene models. A total of 554 136 simple sequence repeats was identified in the whole genome of *C.japonicus*, and dinucleotide microsatellite motifs were the most abundant, accounting for 59.49%. Phylogenomic analysis of 16 genomes based on the 694 single-copy genes suggests that *C.japonicus* is closely related with *Anarrhichthysocellatus*, *Cebidichthysviolaceus*, and *Pholisgunnellus*. The results provide more thorough genetic information of *C.japonicus* and a theoretical basis and reference for further genome-wide analysis.

## ﻿Introduction

*Chirolophis* Swainson, 1839 belongs to the family Stichaeidae of the order Perciformes, which is widely distributed between cold and temperate areas in the Pacific Ocean and along the coasts of Europe in the Atlantic Ocean (Jing et al. 2005; Balanov et al. 2020). *Chirolophis* contains nine species (https://www.fishbase.se/search.php) which are important commercial bony fishes, especially in China (Chen et al. 2017). Among these species, *Chirolophisjaponicus* (Herzenstein, 1890), also known as *Azumaemmnion* (Jordan & Snyder, 1902), lives in rocky shallow coastal waters of the Pacific Ocean, including the Yellow Sea, the Bohai Sea, the northern Sea of Japan, and the Okhotsk Sea to the Bering Sea (Shiogaki 1983; Jing et al. 2005; Balanov et al. 2020). They display strong cryptic habits and are almost impossible to be observed by SCUBA diving observations. Studies on this species are relatively rare, mainly including mitochondrial genome data (Yang et al. 2016), the origin of the cortical protrusion of head (Sato 1977), and reproductive biology research (Chen 2017).

Genome-based phylogenetic studies have provided new opportunities for exploring the phylogeny of fishes. With the development of molecular biology and sequencing technology, more and more species are being sequenced and genomes published, ranging from model fishes to many commercial species. There are nearly 9900 species published genomes in the Eukaryota on the NCBI database (https://www.ncbi.nlm.nih.gov/genome/), accessed on 7 July 2022. Genome survey sequencing (GSS) was considered useful for providing basic genome information. Besides productively identifying genome-wide simple sequence repeats (SSRs) effectively, it can predict putative gene functions efficiently and target the potential exon-intron boundaries. A series of research advances has been made in the study of phylogenomic relationships of organisms, such as plants (Ran et al. 2018; Li et al. 2019b), animals (Koepfli et al. 2015; Heras et al. 2020), and fungi (Spatafora et al. 2016; Liu et al. 2022), which have provided insight into evolutionary history.

In the order Perciformes, the genomes of only three species, *Anarrhichthysocellatus* (Ayres, 1855), *Cebidichthysviolaceus* (Girard, 1854), and *Pholisgunnellus* (Linnaeus, 1758), have been published so far (Li et al. 2019a; Heras et al. 2020; Potter and Consortium 2022). Meanwhile, the complete mitogenomes of two species, *Chirolophisascanii* and *Chirolophisjaponicus* (or *Azumaemmnion*), provided robust phylogenetic relationships (Yang et al. 2016; Chen et al. 2017; Margaryan et al. 2021). Completed genome sequences of *C.japonicus* would improve our understanding of phylogeny, even though the genomic information of *C.japonicus* remains unknown.

In this present study, we perform a genomic survey for *C.japonicus* using next-generation sequencing technology for the first time, investigate its genomic feature and reconstruct the phylogenomic relationships with single-copy orthologs genes of *C.japonicus*. The draft genome assembly of *C.japonicus* can help us find more useful information for taxonomic studies, adaptive evolutionary mechanisms, and phylogenetic studies, as well as understand the genomic evolution of *Chirolophis*, and provide a molecular basis of *C.japonicus*.

## ﻿Materials and methods

### ﻿Material collection

**Figure 1.**
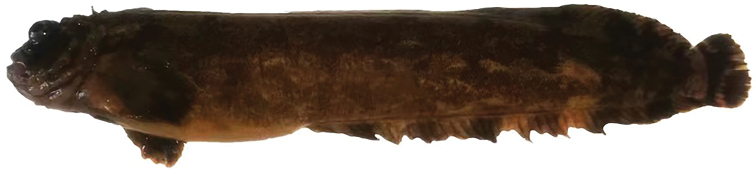
*Chirolophisjaponicus* (Herzenstein, 1890), 186 mm, from Qingdao.

In this study, a male specimen of *C.japonicus* with body length 186 mm and body weight 225 g was collected from coastal waters of Qingdao (35°40'N, 119°30'E), China in July 2021 (Fig. 1). Firstly, we identified it by morphological characteristics and DNA barcoding (mitochondrial DNA COI gene), then the examined sample was quickly preserved in −80 °C ultra-low temperature freezer. All subsequent animal experiments took place at Fisheries Ecology and Biodiversity Laboratory (**FEBL**) of Zhejiang Ocean University, Zhoushan, China. Experiments were conducted under the guidelines and approval of the Ethics Committee for Animal Experimentation of Zhejiang Ocean University (ZJOU-ECAE20211876). Secondly, a piece of fresh muscle tissue was clipped from the base of dorsal fin and preserved in absolute 95% ethanol.

### ﻿Genomic DNA extraction and next-generation sequencing

The total cell DNA was extracted using the phenol-chloroform method (Sambrook et al. 1982), following the protocol in a previous study (Yang et al. 2021), and then carried out with DNA/Protein Analyzer and 1% agarose gel electrophoresis. High-quality DNA was randomly interrupted using ultrasonic crusher, and the obtained short reads (300–350 bp) were sequenced with Illumina NovaSeq 6000 with a paired-end library following the manufacturer’s instructions (OneMore-Tech, Wuhan, China) in January 2022.

### ﻿Sequence quality control, genome assembly, and K-mer analysis

Quality control was performed on the raw data from the Illumina sequencing platform using the FastQC v. 0.11.9 (Andrews 2010) and Trimmomatic v. 0.39 (Bolger et al. 2014) based on four criteria: 1) removal of the A-tail and adaptors, 2) deletion of the low-quality reads where N contents are more than 10%, 3) filtration of the reads whose base quality is less than 10, and 4) discard of duplicated reads. The genome size, heterozygosity, and repeat content of *C.japonicus* was estimated based on a K-mer method (Liu et al. 2013). De novo assembly of the *C.japonicus* genome was conducted using MaSuRCA v. 3.3.3 (Zimin et al. 2013) based on clean data. The quality of the assembled genome was evaluated by Quast v. 5.0.2 and BUSCO v. 5.3.2 (Simão et al. 2015). The mitochondrial DNA analyses followed the method of previous studies (Yang et al. 2016, 2021; Nie et al. 2021). In brief, the software NOVOPlasty v. 4.2.1 (Dierckxsens et al. 2017) and GetOrganelle v. 1.7.6.1 (Jin et al. 2020) were used to assemble the mitogenome with clean data. The mitogenome of *C.japonicus* was annotated using MFannot tool (http://megasun.bch.umontreal.ca/cgi-bin/mfannot/mfannotInterface.pl) and GeSeq (Tillich et al. 2017), then manually annotated and drawn with OGDraw v. 1.3.1 (Lohse et al. 2013; Greiner et al. 2019). The clean data and complete assembled mitochondrial genome were uploaded to GenBank.

### ﻿Gene prediction and functional annotation

The gene predictors Augustus v. 3.3.3 (Stanke and Waack 2003), SNAP (Johnson et al. 2008), and GeneMark-ES v. 4.69 (Lomsadze et al. 2005) were trained on the gene models, and all the gene models were integrated using EvidenceModeler v. 1.1.1 (Haas et al. 2008). The amino acid sequences from *C.japonicus* were annotated by GO (Ashburner et al. 2000), Eggnog (Huerta-Cepas et al. 2019), CAZymes (Cantarel et al. 2009), InterPro (Hunter et al. 2009), KEGG (Kanehisa et al. 2006), KOG https://www.creative-proteomics.com/services/kog-annotation-analysis-service.html, and Pfam (El-Gebali et al. 2019), using Diamond v. 2.0.2 with the e-value less than 1 × 10^−5^ (Buchfink et al. 2015).

### ﻿Microsatellite identification and non-coding RNA annotation

In this study, MIcroSAtellite identification tool (MISA) v. 2.1 was used to identify simple sequence repeats (SSR) in the draft genome of *C.japonicus* (Thiel et al. 2003). The tRNA and rRNA were predicted by tRNAscan-SE v. 3.0 (Lowe and Eddy 1997) and RNAmmer v. 1.2 (Lagesen et al. 2007), respectively.

### ﻿Phylogenomic analysis of *C.japonicus*

A total of 15 genomes of other bony fish were downloaded from the NCBI database (Table 1). The amino acid sequences of single-copy orthologs genes among the 16 species were found using OrthoFinder v. 2.5.4 (Emms and Kelly 2019), and these sequences were aligned by using MAFFT v. 7 (Katoh and Standley 2013). In order to reconstruct the phylogenomic relationship of *C.japonicus*, a maximum likelihood (ML) tree was analyzed/constructed using RaxML v. 8.2.12 based on the amino acid sequences of single-copy orthologs genes (Stamatakis 2014). The best model was PROTGAMMAILGF with 100 bootstrap replicates. Finally, the phylogram was viewed using FigTree v. 1.4.4 (http://tree.bio.ed.ac.uk/software/figtree/).

**Table 1.** Information on genomes used in this study.

Species	Biosample	Bioproject	References
*Anarrhichthysocellatus*	SAMN10245424	PRJNA496475	
*Archocentruscentrarchus*	SAMN09948522	PRJNA489129	Koepfli et al. 2015
*Cebidichthysviolaceus*	SAMN06857690	PRJNA384078	Heras et al. 2020
***Chirolophisjaponicus***			**This study**
*Cyclopteruslumpus*	SAMN12629502	PRJNA625538	
*Gasterosteusaculeatus*	SAMN15223905	PRJNA707557	Berner et al. 2019; Nath et al. 2021
*Gymnodracoacuticeps*	SAMEA104242997	PRJEB37639	
*Liparistanakae*	SAMN10970109	PRJNA523297	
*Micropterussalmoides*	SAMN15299117	PRJNA687018	Broughton and Reneau 2006; Sun et al. 2021
*Myoxocephalusscorpius*	SAMEA4028818	PRJEB12469	
*Pholisgunnellus*	SAMEA7522838	PRJEB45449	
*Pseudoliparis* sp.	SAMN10662039	PRJNA512070	Mu et al. 2021
*Seriolalalandi*	SAMN04902367	PRJNA319656	Purcell et al. 2018
*Taurulusbubalis*	SAMEA7522994	PRJEB45317	
*Toxotesjaculatrix*	SAMN18445299	PRJNA723051	
*Ophiodonelongatus*	SAMN13559843	PRJNA595583	Longo et al. 2020

## ﻿Data availability statement

Raw sequencing data for genome have been deposited at the Sequence Read Archive SRR21530970. These data can be quickly accessed by checking the project ID PRJNA879413 at NCBI Project.

## ﻿Results

### ﻿Sequencing data statistics and K-mer analysis

In this study, a total of 65.4 Gb clean reads was obtained by next-generation sequencing from an Illumina NovaSeq 6000 platform. The Q20 value, Q30 value, and GC content were 98.17%, 94.83%, and 43.14%, respectively. The K-mer analysis with a depth of 71 shows that genome size of *C.japonicus* was 596 Mb with 0.50% heterozygosity rate and 30.30% repeat sequences (Table 2, Suppl. material 1), resulting in *C.japonicus* being a diploid.

**Table 2.** The genome characteristics of *Chirolophisjaponicus* based on the K-mer method.

Species	K-mer number	K-mer depth	Genome size (Mb)	Heterozygous ratio (%)	Repeat sequences (%)
*C.japonicus*	4.353×10^10^	71	596	0.50	30.30

### ﻿Genomic and mitochondrial features

The genome sequences of *C.japonicus* were sequenced from a male with an Illumina NovaSeq 6000 platform, spanning 595.7 Mb with GC contents of 42.61% that were assembled using the software MaSuRCA (Table 3; Zimin et al. 2013). A total of 49 901 contigs was generated with the largest contigs of 365 029 bp. The final contigs N50 and L50 were 29 108 bp and 5388 bp long, respectively (Table 3). A total of 69 rRNA was identified, including 66 8S rRNA, two 18S rRNA, and single 28S rRNA. In addition, 846 tRNA were annotated using the tRNAscan-SE.

**Table 3.** Gene prediction and annotation of *Chirolophisjaponicus*.

Category	Database	Number of reads	Percent (%)
Protein-coding gene model		46 729	
Annotated	InterPro	37 169	79.54
	Eggnog	37 742	80.98
	GO	9353	20.02
	KEGG_KO	17 747	37.98
	Pfam	26 530	56.77
	KOG	35 440	75.84
	CAZymes	765	1.64
Assembly BUSCO coverage			88.9

**Figure 2.**
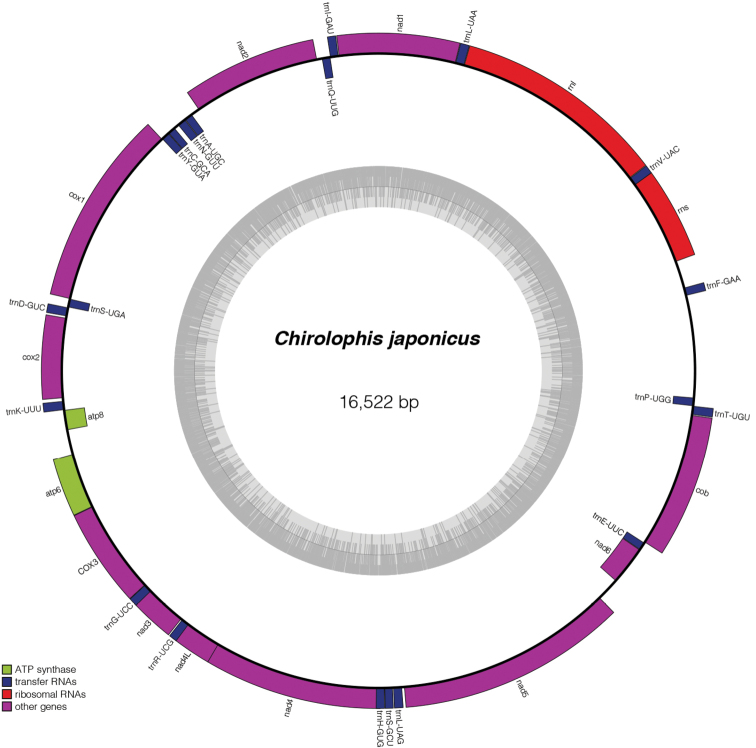
The complete mitogenome structure of *Chirolophisjaponicus*.

The complete mitogenome of *C.japonicus* is 16,522 bp long with a GC content of 45.97%. It consists of two ribosomal RNA genes (rnl and rns), 20 tRNA genes, and 13 protein-coding genes (PCGs) without an intron (Fig. 2).

### ﻿*Chirolophisjaponicus* genome annotation

A total of 46 729 protein-coding genes was predicted by a combination of different software, including Augustus v. 3.3.3 (Stanke and Waack 2003), SNAP (Johnson et al. 2008) and GeneMark-ES v4.69 (Lomsadze et al. 2005). Among these, 79.54%, 80.98%, 20.02%, 39.98%, 56.77%, 75.84%, and 1.64% genes were annotated in the InterPro, Eggnog, GO, KEGG_KO, Pfam, KOG, and CAZymes databases, respectively.

### ﻿Distribution and features of SSR

**Figure 3.**
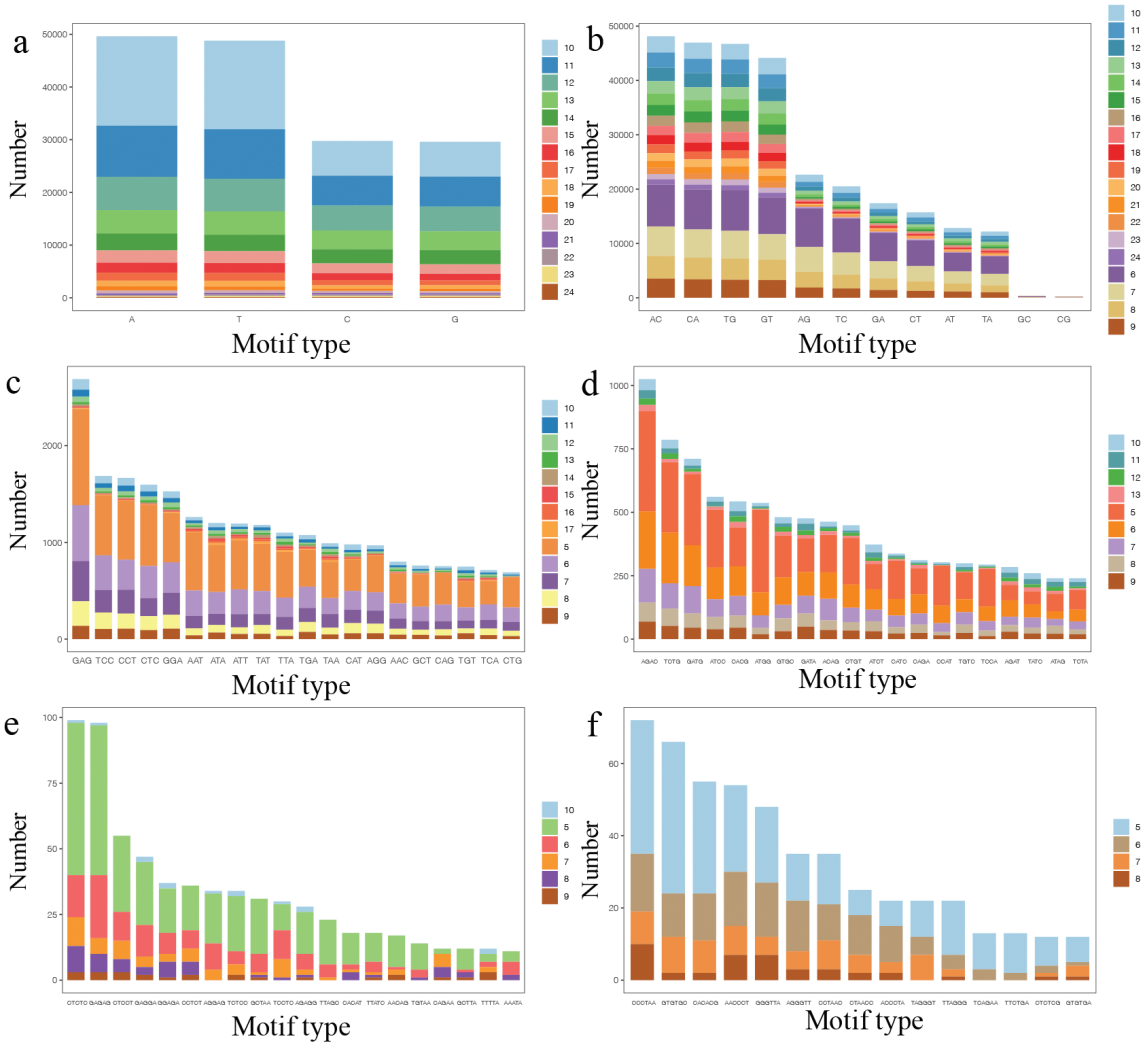
The distributions and frequencies of microsatellite motifs of *Chirolophisjaponicus***a** mononucleotide microsatellite motifs **b** dinucleotide microsatellite motifs **c** trinucleotide microsatellite motifs **d** tetranucleotide microsatellite motifs **e** pentanucleotide microsatellite motifs **f** hexanucleotide microsatellite motifs.

A total of 554 136 of SSR was identified in the complete genome of *C.japonicus*, including 166 077 of mononucleotide microsatellite motifs (29.97%), 329 685 of dinucleotide microsatellite motifs (59.49%), 37 615 of trinucleotide microsatellite motifs (6.79%), 17 896 of tetranucleotide microsatellite motifs (3.23%), 1568 of pentanucleotide microsatellite motifs (0.28%), and 1322 of hexanucleotide microsatellite motifs (0.24%;) (Fig. 2). A/T, AC, GAG, AGAC, CTCTC, and CCCTAA were the highest repeats in mono-, di-, tri-, tetra-, penta-, and hexanucleotide microsatellite motifs, respectively (Fig. 3).

### ﻿Phylogenomic relationships of *Chirolophisjaponicus*

**Figure 4.**
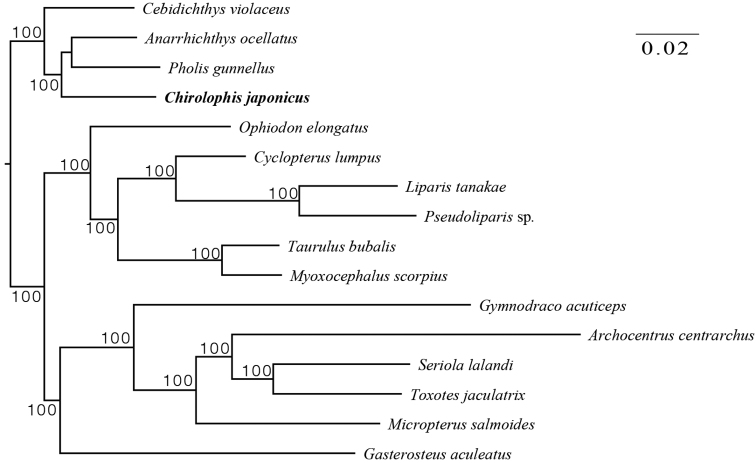
A maximum likelihood (ML) phylogenomic tree of *Chirolophisjaponicus* based on amino acid sequences of 694 single-copy genes. *Chirolophisjaponicus* is in bold. Maximum likelihood bootstrap values (90%) of each clade are indicated along branches. A scale bar in the upper right indicates substitutions per site.

**Figure 5.**
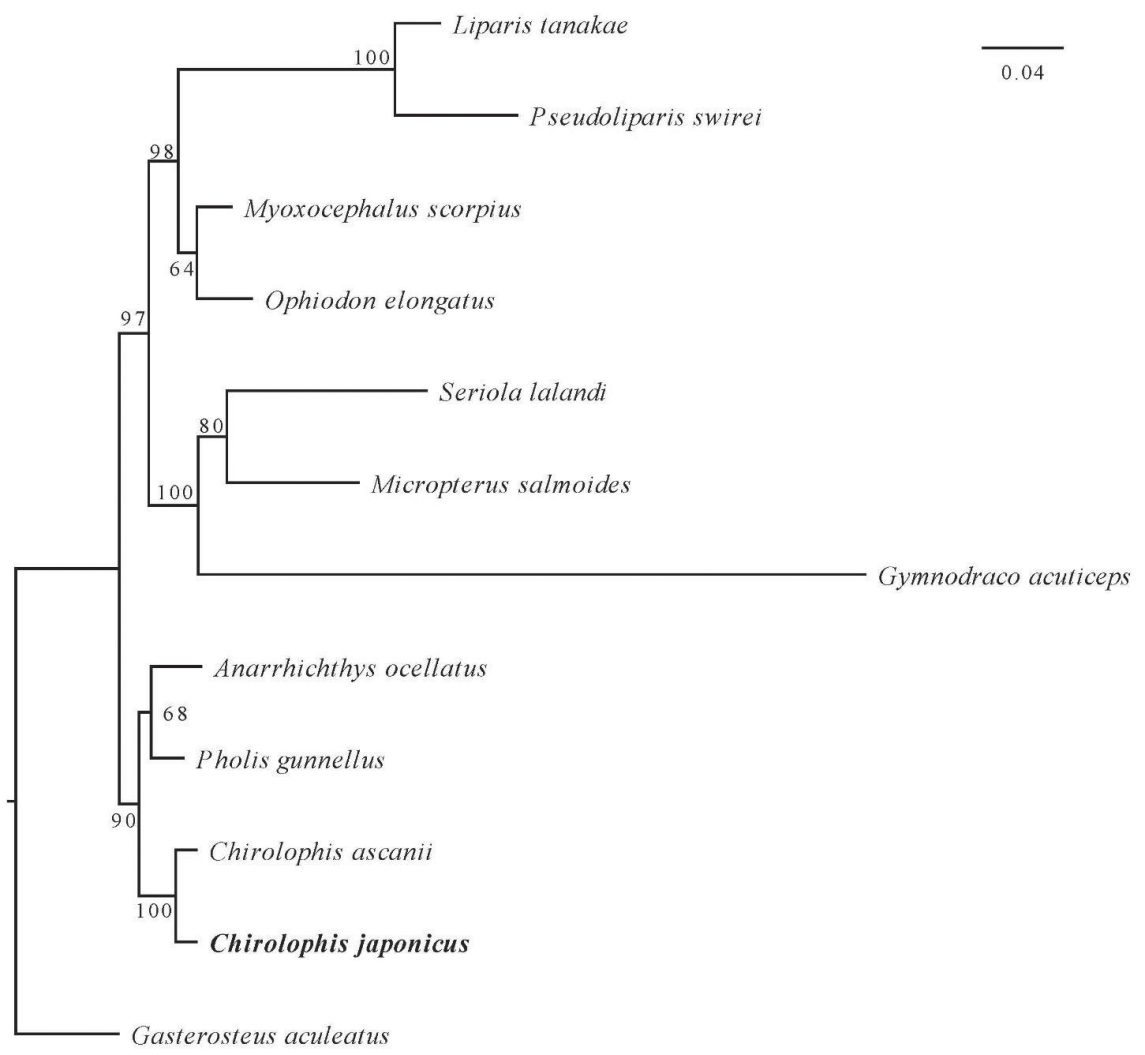
The maximum likelihood (ML) phylogenomic tree of fungi based on amino acid of 13 protein-coding genes (PCGs): ATP6, ATP8, COX1, COX2, COX3, CYTB, ND1, ND2, ND3, ND4, ND4L, ND5 and ND6. Support values for ML analysis greater than 60% is given on relative clade. A scale bar in the upper left indicates substitutions per site.

In the present study, the phylogenomic relationship of a total of 16 bony fish (Table 1, Fig. 4) was reconstructed. A total of 694 single copy genes (Suppl. material 2) was identified from 16 male fish genomes using OrthoFinder (Emms and Kelly 2019), which consisted of 361 031 characters from amino acid sequences. The phylogenomic tree suggested that *Chirolophisjaponicus* is closely related with *Anarrhichthysocellatus*, *Cebidichthysviolaceus*, and *Pholisgunnellus*, and provided robust phylogenetic relationships within the order Zoarcales, with full support. Although *Chirolophisjaponicus* and *Cebidichthysviolaceus* belong to the family Stichaeidae, they did not form a clade based on the amino acid sequences of 694 single-copy genes. In addition, *Ophiodonelongatus*, *Cyclopteruslumpus*, *Liparistanakae*, *Pseudoliparis* sp., *Taurulusbubalis*, and *Myoxocephalusscorpius* clustered into a clade; other species, including *Gymnodracoacuticeps*, *Archocentruscentrarchus*, *Seriolalalandi*, *Toxotesjaculatrix*, *Micropterussalmoides* and *Gasterosteusaculeatus*, formed a separate clade. In addition, the phylogenomic analysis based on the amnio acid of 13 protein-coding genes of mitogenome show that *Chirolophisjaponicus* is closely related with the *Chirolophisascanii* (Fig. 5).

## ﻿Discussion

Currently, there are more than 30 000 species of fishes, including bony, jawless, and cartilaginous fishes, living on the earth, some with great ecological and economic value. In 2002, the first fish genome, *Fugurubripes* (also known as “torafugu”) was published, which provided a framework for future studies of fish genomes (Aparicio et al. 2002). With the rapid development of the whole genome sequencing (WGS) technology, a large number of fish genomes have since been sequenced, such as fish as model organisms *Oryziaslatipes* and *Daniorerio*, and economically important fishes such as *Cyprinuscarpio* and *Ctenopharyngodonidella* (Kasahara et al. 2007; Howe et al. 2013; Xu et al. 2014; Wu et al. 2022). In addition, the Chinese “Aquatic 10-100-1000 Ge- nomics Program” and the “Fish 10K Project” have facilitated the understanding of fish genomes (Liu et al. 2017; Fan et al. 2020). Until now, a total of 819 fish genomes has been released in the NCBI database (https://www.ncbi.nlm.nih.gov/genome/, assessed on 7 July 2022), which is less than3% of the known 30 000 species.

In the present study, a new fish genome, *Chirolophisjaponicus*, was sequenced. The genomes size was estimated to be 596 Mb based on the K-mer analysis, and the genome spanned 595.7 Mb, assembled using the MaSuRCA (Table 3; Zimin et al. 2013), which followed the predicted genome size of the K-mer method. Among the published teleost genomes, the size ranges from 322.5 Mb (*Fugurubripes*) to 40 Gb (*Protopterusannectens*) (Aparicio et al. 2002; Wang et al. 2021), with an average length less than 1 Gb (Fan et al. 2020). Meanwhile, the genome of three species in Zoarcales, including *Anarrhichthysocellatus* (612.19 Mb), three genomes of *Cebidichthysviolaceus* (575.66 Mb, 593.00 Mb, 606.18 Mb), and two genomes of *Pholisgunnellus* (588.7 Mb, 590.3 Mb), are slightly larger than that of *C.japonicus* (https://www.ncbi.nlm.nih.gov/genome/, assessed on 7 July 2022). In addition, the heterozygous ratio of *C.japonicus* was 0.50%, probably mid-level compared to other teleost genomes (Aird et al. 2011; Li et al.2019c; Xu et al. 2020; Yang et al. 2021).

At present, phylogenomic analysis has become an important method for studying the evolutionary relationships of an organism, such as plants (Ran et al. 2018; Li et al. 2019b), animals (Koepfli et al. 2015; Heras et al. 2020), and fungi (Spatafora et al. 2016; Liu et al. 2022). Although the phylogenetic relationships of the genus *Chirolophis* have been published based on the mitogenomes (Yang et al. 2016; Chen et al. 2017; Margaryan et al. 2021), we provided a phylogenomic relationship according to the 694 single-copy genes (Fig. 3, Suppl. material 2) among *C.japonicus* and 15 other species. The results of the phylogenomic tree shows that *C.japonicus* is closely related with three species in the order Zoarcales, while *C.japonicus* and *Cebidichthysviolaceus*, belonging to the family Stichaeidae, are without a clade (Fig. 2). Thus, solving this problem requires more fish genomes to be sequenced.

Microsatellite DNA markers shows many advantages, such as codominant, extensive distribution, abundant polymorphisms, and a convenient analysis, and was considered to be an effective tool in genetic analysis and evolutionary research (Yang et al. 2022). In this study, the highest number and type of repeats is dinucleotide repeats, which was consistent with data for *Ophichthusevermanni* (Yang et al. 2022), *Padonnehereus* (Yang et al. 2021), *Cociellacrocodilus* (Zhao et al. 2021), *Acanthogobiusommaturus* (Chen et al. 2020), *Sillagosihama* (Qiu et al. 2020), and other species. SSR polymorphic loci are mainly distributed among mononucleotide and dinucleotide repeats. Based on this, the search of polymorphic SSR markers from low repetitive motifs will greatly help in subsequent population genetics research of *C.japonicus*. The complexity of repeated motif usually reflects DNA mutation rate and evolutionary level (Katti et al. 2001). The frequency from mononucleotides to trinucleotides was up to 96.25%, which implies that *C.japonicus* has experienced a long evolutionary history and accumulated more genetic variation.

Finally, the genome assembly of *C.japonicus* can help us understand the genome evolution of *Chirolophis* and teleosts, as well as provide a molecular basis for breeding and cultivation.

## Supplementary materials

Supplementary material 1 K-mer analyses (K = 71) of *Chirolophisjaponicus*, X-axis and Y-axis represent the K-mer depth and frequency for the corresponding depth Data type image https://binary.pensoft.net/file/768512This dataset is made available under the Open Database License (http://opendatacommons.org/licenses/odbl/1.0/). The Open Database License (ODbL) is a license agreement intended to allow users to freely share, modify, and use this Dataset while maintaining this same freedom for others, provided that the original source and author(s) are credited.Lu Liu, Qi Liu, Tianxiang Gao

Supplementary material 2 Maximum likelihood phylogenomic tree of *Chirolophisjaponicus* Data type phylogenomic tree Explanation note Maximum likelihood phylogenomic tree of *Chirolophisjaponicus* based on amino acids of 694 single-copy genes. https://binary.pensoft.net/file/768513This dataset is made available under the Open Database License (http://opendatacommons.org/licenses/odbl/1.0/). The Open Database License (ODbL) is a license agreement intended to allow users to freely share, modify, and use this Dataset while maintaining this same freedom for others, provided that the original source and author(s) are credited.Lu Liu, Qi Liu, Tianxiang Gao
