# Supplementary figures and images for: ﻿Genome-wide survey reveals the phylogenomic relationships of Chirolophisjaponicus Herzenstein, 1890 (Stichaeidae, Perciformes)
Source: Zookeys. 2022 Nov 11;1129:55–72. doi: 10.3897/zookeys.1129.91543 (PMC9836534; doi:10.3897/zookeys.1129.91543)

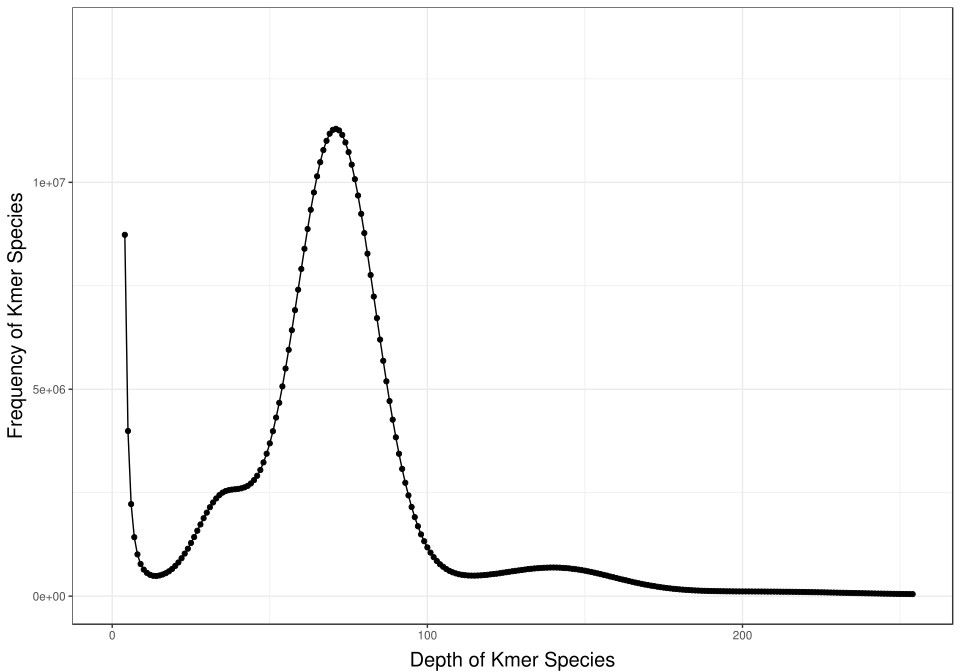

Supplement: Supplementary material 1 — K-mer analyses (K = 71) of Chirolophisjaponicus, X-axis and Y-axis represent the K-mer depth and frequency for the corresponding depth [file zookeys-1129-055_article-91543__-s001.png]
